# The Incidence of Abnormal Preoperative Testing Among Elective Surgery Patients in a Referral Hospital in Southern Saudi Arabia

**DOI:** 10.7759/cureus.57448

**Published:** 2024-04-02

**Authors:** Abdulmajeed Alkhathami, Ahmed Alameer, Osama A Alqarni, Amal T Aljuaid, Elaf S Alrimthi, Saeed Nasser A Alaklabi, Mutasim E Ibrahim

**Affiliations:** 1 Ophthalmology, College of Medicine, University of Bisha, Bisha, SAU; 2 Breast and Endocrine Surgery, University of Bisha, Bisha, SAU; 3 College of Medicine, University of Bisha, Bisha, SAU; 4 Microbiology, College of Medicine, University of Bisha, Bisha, SAU

**Keywords:** kingdom of saudi arabia (ksa), retrospective, elective surgeries, abnormal findings, routine preoperative testing

## Abstract

Background

Preoperative investigations are important to assess the clinical condition of patients who undergo elective surgical procedures. However, there is still debate about the usefulness of performing preoperative investigations. We aimed to determine the prevalence of routine preoperative investigation abnormalities among elective general surgery patients.

Methodology

This retrospective hospital-record-based study was conducted at the King Abdullah Hospital in Bisha Province, southern Saudi Arabia. General and clinical data of 968 patients who underwent elective surgical interventions from February 2022 to January 2023 were retrieved and analyzed.

Result

A total of 968 patients (578 females and 390 males) aged between 14 and 80 years were included in the study. Four hundred and eleven (42.5%) patients were in the age group of 40 years and above. The commonly detected comorbidities among the patients were diabetes (15%), hypertension (12%), respiratory diseases (7.5%), and cardiac diseases (2.5%). Abnormalities related to hemoglobin (31%), total leucocyte count (12.7%), and platelets (8.5%) were found in 968 patients. Around 15% of patients had increased creatinine levels. Hypokalemia was observed in 6.8% of patients. Increased liver enzymes were reported in limited proportions (10% to 14%) of patients. Slightly abnormal radiological findings were reported for chest X-ray (CXR) (2.8%), electrocardiogram (ECG) (2%), and Doppler echocardiography (Echo) (0.8%). A statistically significant association between the age of the patient and abnormal findings of ECG (p <.001), Echo (p = .001), and CXR (p <.001). Cardiac abnormalities were commonly associated with patients of ≥ 40 years. Abnormal cardiac findings (CXR, ECG, Echo) were significantly (p<.05) increased with the presence of diabetes, hypertension, and cardiovascular comorbidities.

Conclusions

Preoperative testing revealed limited proportions of abnormal findings among patients with elective surgical procedures. Routine ordering of many preoperative investigations without specifications may not predict postoperative complications of the patients. Therefore, undertaking preoperative testing should be guided by targeted history, physical examination, clinical risk factors, and type of surgical procedure intended to be performed.

## Introduction

Elective surgery is the most commonly performed operation in hospital settings around the globe [1-3]. In the United States, the number of patients requesting elective surgery has increased, representing a significant proportion of healthcare costs. Over 36 million procedures are performed annually, and 22.5 million in ambulatory surgery [3].

Preoperative investigations are important to assess the clinical conditions of patients who undergo elective surgical procedures [4]. These investigations are essential to detect asymptomatic diseases and identify potential conditions that can affect surgical and anesthetic outcomes [4,5]. It can also provide comprehensive information that promotes patient safety and decreases the liability for harmful incidents [4-7]. Preoperative investigations are generally classified into two groups, mainly routine and diagnostic tests. Regular preoperative examinations requested in the absence of any specific clinical signs or purpose are intended to discover diseases or disorders in asymptomatic patients [2]. These groups of preoperative investigation usually include a panel of blood, urine tests, chest X-ray (CXR), electrocardiogram (ECG), and Doppler echocardiography (Echo) [2,8].

In the literature, there is still debate about the usefulness of performing preoperative investigations [2,9]. A previous study indicated that preoperative investigations of hemoglobin and hematocrit, glucose, blood urea nitrogen, creatinine, electrocardiogram, and chest radiograph should be routinely ordered for healthy older patients of age more than 60 years. In addition, selective ordering of investigations should be undertaken based on clinical findings and comorbid conditions [7]. Other studies claimed that the practice of routine investigation is costly and leads to unnecessary delays of surgeries [10-12]. However, detecting abnormalities may have no impact or significant effect on the surgery outcome and may lead to further irrelevant studies [10,13].

There is insufficient evidence that surgical outcomes and postoperative complications are changed or predicted by routine preoperative investigations [14]. An earlier study in Kuwait found that 14.1% of preoperative tests were abnormal, 9.2% of which were expected, and only 4.9% were unexpected. However, no related complications or alterations in preoperative care have been recorded in patients with unforeseen abnormalities [12]. A recent systematic review found that preoperative testing based on the patient's clinical condition has financial benefits, without compromising patient safety and quality of healthcare [8]. Although many studies demonstrated the usefulness and cost-effectiveness of preoperative tests without clinical indication [2,15], these investigations are still routinely performed in many Saudi hospitals [16-18]. In addition, studies estimating the prevalence of abnormal preoperative testing of elective surgical patients in Saudi Arabia are scarce. This study aims to determine the prevalence of routine preoperative investigation abnormalities among patients of elective general surgery in King Abdullah Hospital in Bisha Province, southern Saudi Arabia. Evaluating the level of abnormal preoperative investigations of patients undergoing elective surgery is important to understand their efficiency. It thus might help set up guidelines and strategies for preoperative testing.

## Materials and methods

This retrospective, hospital-record-based study was carried out at the King Abdullah Hospital (KAH) in Bisha Province in the Aseer region, southern Saudi Arabia. KAH is a main referral hospital with a 400-bed capacity serving a large catchment area of Bisha and neighboring provinces in the Aseer region. Over the first six months of 2022, KAH has served 421.968 patients, performing 1.410 surgeries and 1.175 one-day surgeries [19].

Ethical approval

The study was approved by the Research Ethics Local Committee at the College of Medicine, University of Bisha, Saudi Arabia (UB-RELOC H-06-BH-087/ (0605.23), and it followed the ethical standards of the Helsinki Declaration. Informed consent was not obtained for this study, as the patients' data were retrieved from the hospital's electronic database. Patients' confidentiality was kept, as the data collection sheet had no personal data referring to or implying the patients' identity.

General and clinical data of 968 patients who underwent elective surgical interventions from February 2022 to January 2023 were collected and analyzed. The data were retrieved from the patient's electronic files and the hospital's electronic database. It was collected anonymously without violating patients' identities. These include sociodemographic characteristics, types of comorbidities, and findings of ECG, CXR, Echo, and routine laboratory tests. The laboratory investigations included renal function tests (creatinine, sodium, and potassium) and complete blood count (total leucocyte counts (TLC), neutrophil counts, hemoglobin concentration, and platelet counts), and liver function tests (alanine transaminase (ALT), aspartate aminotransferase (AST), gamma-glutamyl transferase (GGT), alkaline phosphatase (ALK)), and serological screening of hepatitis B virus (HBV) and hepatitis C virus (HCV). The study included all patients selected for elective surgery in the general surgery department during the study period. Patient files with missed data or incomplete information were excluded from the study.

We defined routine baseline investigations as those minimal tests administered to all patients regardless of disease, clinical findings, or comorbidities [7,8]. Laboratory test results were reported as normal and abnormal per their standard reference values [20,21].

Statistical methods

Data were collected, arranged, and entered in a Microsoft Excel sheet (Microsoft Corporation, Redmond, WA, USA) and then transferred to IBM SPSS version 25.0 (IBM Corp, Armonk, NY, USA). Descriptive statistics were presented as mean, standard deviation, proportions, and frequency tables. The chi-square and Fisher exact tests were used, when appropriate, to compare each two variables. P values of less than 0.05 were considered statistically significant.

## Results

A total of 968 patients aged between 14 and 80 years were involved in the study. Of these, 578 (59.7%) were females, and 390 were males. Those aged less than 40 years were 57.5% (n=557), and 76.7% (n=742) were married. About 15% (n=148) were diabetic patients and 12% (n=105) had hypertension. Respiratory diseases were present in 7.5% (n=73), and cardiac diseases were present only in 2.5% (n=24). Most of the patients had no previous history of surgery (70.6%; n=683). Detailed baseline and clinical characteristics of the patients are presented in Table 1.

**Table 1:** Baseline characteristics of the patients

Parameter	n (%)
Age (years)	
<40	557 (57.5%)
≥40	411 (42.5%)
Gender	
Male	390 (40.3%)
Female	578 (59.7%)
Marital status	
Single	226 (23.3%)
Married	742 (76.7%)
Diabetes mellites	
Yes	148 (15.3%)
No	820 (84.7%)
Hypertension	
Yes	105 (10.8%)
No	863 (89.2%)
Cardiac disease	
Yes	24 (2.5%)
No	944 (97.5%)
Respiratory disease	
Yes	73 (7.5%)
No	895 (92.5%)
Previous surgery	
Yes	285 (29.4%)
No	683 (70.6%)

Table 2 shows the results of the routine preoperative laboratory investigations of the patients (n=968). Regarding CBC, abnormal findings were reported among patient blood samples for neutrophil count in 313 (32.3%) patients, decreased hemoglobin in 300 (31%), and platelet counts in 82 (8.5%) patients. Liver function tests showed abnormal values in 134 (13.8%) patients for ALT, 125 (12.9%) patients for GGT, and 100 (10.3%) patients for AST. Renal function tests revealed abnormal values in 148 (15.3%) patients for creatinine and 66 (6.8%) patients for potassium. Viral seropositivity (hepatitis B or C) was detected in seven (0.7%) patients. A limited number of patients showed abnormal findings for CXR (2.8%), ECG (2%), and Echo (0.8%).

**Table 2:** Findings of routine preoperative investigations of elective surgical patients ALK: alkaline phosphatase, ALT: alanine transaminase, AST: aspartate aminotransferase, GGT: gamma-glutamyl transpeptidase

Parameter (normal value)	n (%) abnormal findings
Complete blood count	
Total leukocyte count (4–11 x10^9^/L)	123 (12.7%)
Neutrophil count (2.0–7.0 G/L)	313 (32.3%)
Hemoglobin (12–16 g/dl for female), (13–18 g/dl for male)	300 (31%)
Platelets (130–400 x10^9^/L)	82 (8.5%)
Renal function and electrolytes	
Creatinine (15–115 μmol/L)	148 (15.3%)
Sodium (135–145 mmol/L)	173 (17.9%)
Potassium (3.5–5.0 mmol/L)	66 (6.8%)
Liver enzymes	
ALT (0.0–41 U/L)	134 (13.8%)
AST (0.0–38 U/L)	100 (10.3%)
GGT (0.0–30 IU/L)	125 (12.9%)
ALK (80–350 U/L)	190 (19.6%)
Viral seropositivity	
Hepatitis B or C	7 (0.7%)
Radiological screening	
Electrocardiogram	19 (2%)
Doppler echocardiography	8 (0.8%)
Chest X-ray	27 (2.8%)

Table 3 shows the association between cardiac radiological findings and patients' general and clinical characteristics. Significant associations were found between the age of the patient and findings of ECG (p <.001), Echo (p = 0.001), and CXR (p <.001). Cardiac abnormalities were commonly associated with patients of ≥ 40 years. Abnormal cardiac findings were significantly increased with the presence of DM, hypertension, and cardiovascular diseases. On the other hand, there were no significant associations (p>0.05) between the cardiac evaluation tests and patient genders, marital status, previous surgery, and presence of respiratory diseases.

**Table 3:** Findings of cardiac evaluation tests in relation to general and clinical characteristics of the patients (n=968)

Factor	Total number	Abnormal electrocardiogram		Abnormal echocardiography		Abnormal chest X-ray	
		n (%)	p-value	n (%)	p-value	n (%)	p-value
Age			< .001>		.001		< .001>
<40	557	1.0 (0.2)		0 (0.0)		5 (0.9)	
≥40	411	18 (4.4)		8 (1.9)		22 (5.4)	
Gender			.155		.721		.552
Male	390	11 (2.8)		4 (1.0)		9 (2.3)	
Female	578	8 (1.4)		4 (0.7)		18 (3.1)	
Marital status			.273		.398		.650
Single	226	2 (0.9)		0 (1.3)		5 (2.2)	
Married	742	17 (2.3)		5 (0.7)		22 (3.0)	
Diabetes			< .001>		.022		.004
Yes	148	10 (6.8)		4 (2.7)		10 (6.8)	
No	820	9 (1.1)		4 (0.5		17 (2.1)	
Hypertension			< .001>		.06		.001
Yes	105	95 (9.5)		4 (3.8)		9 (8.6)	
No	863	854 (1.0)		4 (0.5)		18 (2.1)	
Cardiovascular disease			< .001>		< .001>		< .001>
Yes	24	10 (41.7)		5 (20.8)		7 (29.2)	
No	944	9 (1.0)		3 (0.3)		20 (2.1)	
Respiratory disease			1.0		.476		.256
Yes	73	1 (1.4)		1 (1.4)		0 (0.0)	
No	895	18 (2.0)		7 (0.8)		27 (3.0)	
Previous surgery			.123		.053		.395
Yes	285	9 (3.2)		5 (1.8)		10 (3.5)	
No	683	10 (1.5)		3 (0.4)		7 (2.5)	

Association between findings of preoperative tests and patient characteristics

**Figure 1:**
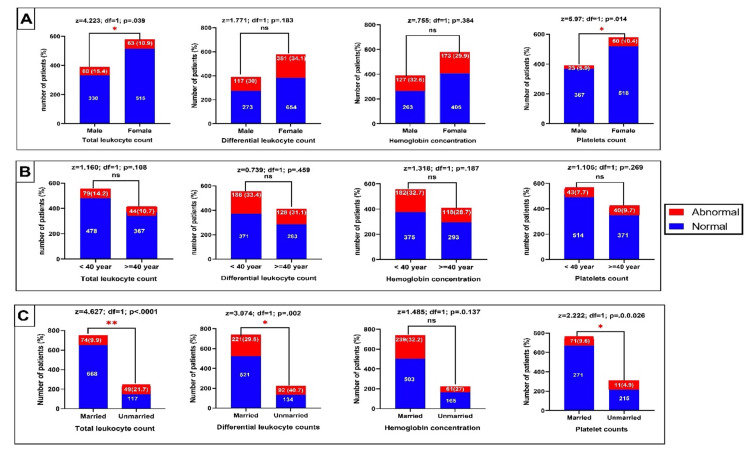
Evaluation of hematological parameters in relation to patient's gender (A), age group (B), and marital status (C) (*) = significance at a P-value less than 0.05; ns = not significant

Figure 1 illustrates the association between hematological findings and patients' general characteristics. TLC was significantly higher among male than female patients (15.4% vs. 10.9%; p=.039). Conversely, platelet counts were significantly higher among female than male patients (10.4% vs. 5.9%; p=.014). On the other hand, there was no statistically significant difference between the patient's age and CBC parameters. Regarding the marital status of the patients, the gender of the patients was statistically significant with total leukocyte count (p= .040). Platelets were not statistically significant with any sociodemographic characteristics.

**Figure 2:**
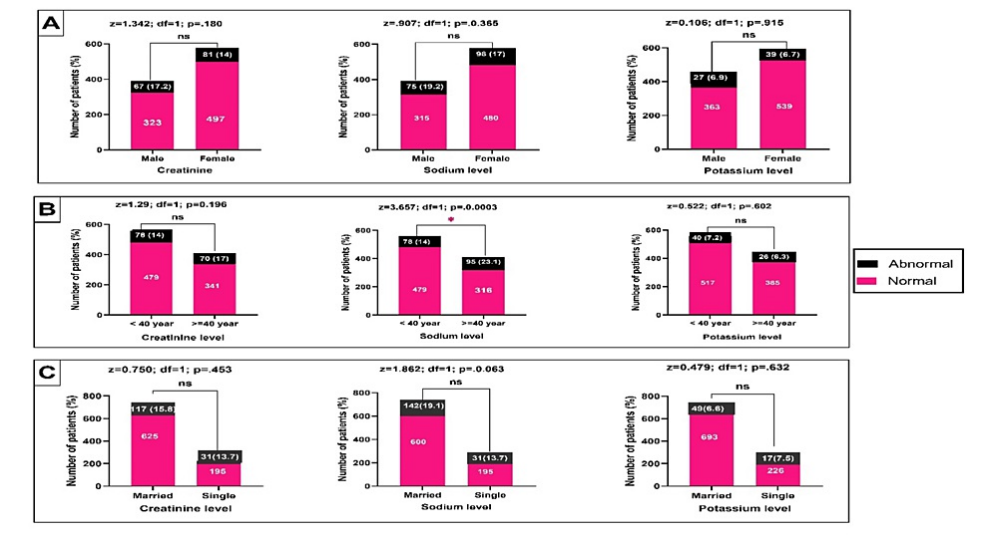
Evaluation of renal function tests in relation to patient's gender (A), age group (B), and marital status (C) (*) = significance at a P-value less than 0.05; ns = not significant

Figure 2 illustrates the renal function tests and their association with the general characteristics of the patients. The patient's age was unrelated to creatinine (p=.508). In contrast, the sodium level was statistically significant with the age of the patients (p < .001).

**Figure 3:**
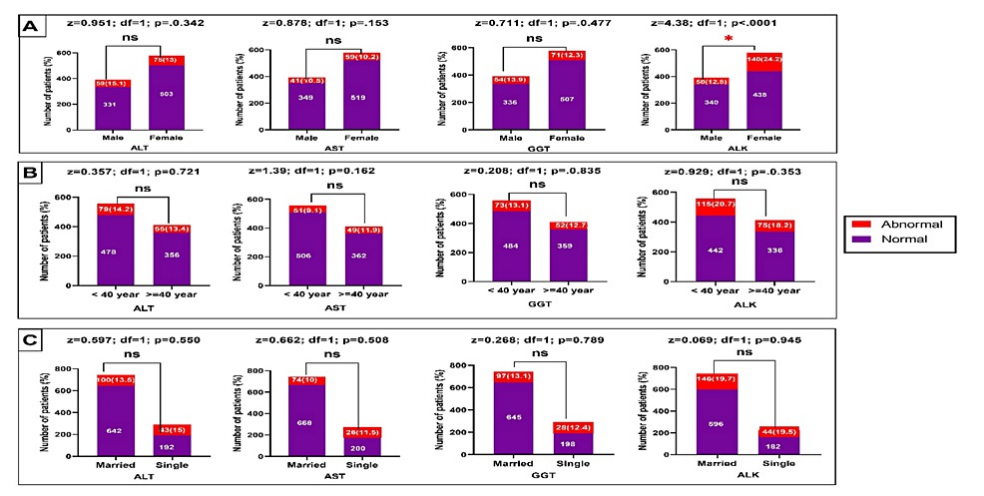
Evaluation of liver enzyme levels in relation to patient's gender (A), age group (B), and marital status (C) (*) = significance at a P-value less than 0.05; ns = not significant

Figure 3 illustrates the findings of liver enzyme tests and the patient's general information. Most sociodemographic characteristics were not associated statically with liver function tests. The history of respiratory disease was statistically significant with GGT (p=0.04).

## Discussion

As a rule, various screening investigations are requested before surgical intervention as baseline values and to find occult diseases and undetected anomalies that could affect the patient in the perioperative setting [14,22]. The list includes laboratory tests of hematology parameters, liver enzymes, renal function tests, serum electrolytes, and viral serology for HBV, HCV, and human immune virus (HIV) infections, in addition to radiological examinations of CXR, ECG, and Echo [6-8]. Not every surgical population will benefit from uniform investigative ordering. The kind and urgency of the surgery (elective, semi-elective, or emergency), the patient's current physiological state, any related co-morbidities, and the drugs are all factors that determine which investigations are needed. When arranging preoperative investigations, the complexity of the surgery is also taken into account. For instance, the National Institute of Clinical Excellence (NICE) classifies surgeries as minor, middle, or complex depending on how invasive they are. Patients undergoing emergency surgery, specialized surgical procedures (such as cardiothoracic, vascular, neurological, or transplant surgery), or those who have a serious systemic illness require individualized investigative ordering [23].

The present study showed that 31% of the patients had abnormal hemoglobin levels during preoperative investigations. This proportion was higher than that reported in other studies [10,24]. For instance, a study in India found that hemoglobin concentration was deceased in 16% of patients undertaking uncomplicated elective surgical procedures [10]. In Pakistan, anemia was detected in 19.2% of patients undergoing ambulatory surgeries [23]. Routine preoperative hemoglobin testing does not have any effect on the perioperative outcomes in asymptomatic patients who are planned for elective daycare surgeries [24]. However, significant blood loss from major surgery is a life-threatening condition [10]. 

Generally, electrolytes and renal function tests are important for patients using diuretics who are exposed to nephrotoxic agents, have heart failure, have pituitary or adrenal disease, or experience major postoperative electrolyte alterations [8,25]. However, serum electrolyte levels are frequently assessed as a part of preoperative testing [7]. Our study found 66 (6.8%) had abnormal potassium. A previous study indicated an insignificant association between electrolyte abnormalities and perioperative morbidity and mortality [18]. Nevertheless, increased potassium concentrations are usually asymptomatic and may lead to life-threatening cardiac arrhythmias [5].

In the present study, the creatinine level was increased in 15% of patients. Consistently, a previous study reported a 12% prevalence of abnormal creatinine in elderly surgical patients [7]. A preoperative serum creatinine level of > 2.0 gm/dl is a significant risk factor for postoperative problems in a revised cardiac index [26]. A recent study documented that renal insufficiency is a predictor of postoperative complications such as myocardial infarction, stroke, pneumonia, septic shock, and postoperative bleeding [27]. Patients with preoperative renal insufficiency have an increased risk of postoperative 30-day mortality and complications [27]. Therefore, examining renal function tests for patients at a higher risk of renal impairment is crucial. It has been suggested that ordering renal function in surgical patients older than 40 years of age is important to adjust the dose of perioperative medications. In addition, renal dysfunction is significantly correlated with poor outcomes in patients undergoing major surgery [24].

In this study, we found a small number of patients have positive serological findings for HCV and HBV. Screening for blood-borne viruses of HBV, HCV, and HIV is crucial to lowering the risk of transmission, but they have no direct impact on perioperative complications [28]. However, their presence can alter the planned procedure or may necessitate postponing surgery or deciding if it is crucial [28].

ECG is regularly performed as one of the critical elements of standard preoperative evaluation and is recommended for patients suspected to have cardiac illnesses [6,8]. Our study found that only 2% of the patients had abnormal ECG findings. It has been recommended that routine ECG is not indicated for the preoperative assessment of patients with no history or symptoms of heart disease and those who are undergoing low-risk surgeries [8]. CXR is one of the most frequent radiological investigations requested in many hospitals [8,22]. In the present study, CXR revealed a limited abnormality in 27 (2.8%) patients, which is in agreement with previous reports [22]. Studies indicated that CXR is an abused test and constituted the highest proportion of unindicated preoperative investigations for elective surgery patients without cardiopulmonary disease [4].

Comparing the findings of ECG, CXR, and Echo with patients' demographics and clinical characteristics, we found a significant association between radiological abnormalities and patients aged ≥ 40 years or older and those with comorbidities (Table 3). It has been documented that patient aged 70 years or older have a 75% chance of having at least one abnormality on their ECG [6]. Therefore, radiological preoperative investigations should be done based on clinical examination and comorbid conditions of patients and in the presence of any risk factors.

In the present study, there was no significant relationship between most sociodemographic factors and preoperative laboratory investigations (Figures 1-3). Nevertheless, patients at ≥ 40 years had significantly higher hypernatremia than those below 40 years. It is well known that elderly patients tend to have more abnormal laboratory test findings than other age groups. Research evidence showed that patients aged 70 years or older have a 10% chance of having abnormal creatinine, hemoglobin, or glucose levels. However, these factors were found not to be predictive of postoperative complications [6].

Study's limitations

This study had a few limitations, as given below.

Single-Center Study

The study's focus on a single referral hospital in southern Saudi Arabia may limit the generalizability of the findings to other hospitals or healthcare settings. Different hospitals may have varying protocols, resources, and patient populations, which could influence the incidence of abnormal properties resetting.

External Factors

The study may not account for external factors that could influence the incidence of abnormal properties resetting such as patient characteristics, comorbidities, or variations in surgical techniques. These factors, if not adequately controlled or accounted for, may confound the study results.

Limited Timeframe

The study might have a limited timeframe for data collection, potentially missing out on long-term complications or incidences occurring beyond the study period. This may not provide a comprehensive understanding of the incidence of abnormal properties resetting among elective surgical patients.

## Conclusions

The study revealed limited proportions of abnormal findings among patients with elective surgical procedures. Routine ordering of such investigations without specifications may not predict postoperative complications of the patients. In addition, it can abuse the number of preoperative tests and costs. Therefore, undertaking preoperative testing should be guided by targeted history, physical examination, clinical risk factors, and type of surgical procedure intended to be performed. However, educating medical practitioners and establishing a clear guideline on whether to order preoperative testing are essential.
